# Comment on 'Hand grip strength‐based cachexia index as a predictor of cancer cachexia and prognosis in patients with cancer' by Xie et al.

**DOI:** 10.1002/jcsm.13298

**Published:** 2023-07-28

**Authors:** Ping'an Ding, Jiaxiang Wu, Haotian Wu, Chenyu Sun, Muzi Meng, Scott Lowe, Yuan Tian, Honghai Guo, Lingjiao Meng, Qun Zhao

**Affiliations:** ^1^ The Third Department of Surgery The Fourth Hospital of Hebei Medical University Shijiazhuang China; ^2^ Hebei Key Laboratory of Precision Diagnosis and Comprehensive Treatment of Gastric Cancer Shijiazhuang China; ^3^ Department of Thyroid and Breast Surgery The Second Affiliated Hospital of Anhui Medical University Hefei China; ^4^ Department of General Surgery the Second Affiliated Hospital of Anhui Medical University Hefei China; ^5^ UK Program Site American University of the Caribbean School of Medicine Preston UK; ^6^ Bronxcare Health System The Bronx NY USA; ^7^ College of Osteopathic Medicine Kansas City University Kansas City MO USA; ^8^ Research Center of the Fourth Hospital of Hebei Medical University Shijiazhuang China

The study conducted by Xie et al.^1^ caught our attention, and we extend our gratitude for their valuable contribution. Measuring skeletal muscle index can be costly and tedious. Xie et al. proposed an alternative by using hand grip strength (HGS) to establish a new composite index, HGS‐based cachexia index (CXI) (H‐CXI), which is calculated as [HGS (kg)/height (m)2 × serum albumin (g/L)]/neutrophil‐to‐lymphocyte ratio. The H‐CXI proved to be a rapid and accurate assessment tool, which can effectively evaluate the risk of cachexia and clinical outcome in cancer patients. The study revealed that H‐CXI is independently associated with patient prognosis and is linked to age, pathological stage, systemic inflammation, and nutritional status. Low H‐CXI was found to be an independent predictor of cachexia and prognosis in cancer patients. Moreover, H‐CXI has shown to be a better prognosis evaluation tool for patients with the same pathological stage. In light of these findings, H‐CXI is an economic and procedural advantage over the original CXI, is a comprehensive indicator, and is a multi‐centre, prospective, large‐sample study that provides reliable results. H‐CXI has a reliable clinical effect and a broad clinical prospect in predicting prognosis and cancer cachexia.

It was found that the study analysed tumours of varying types with different pathological factors, potentially impacting the outcome. Additionally, the study lacked a prospective approach with rigorous inclusion criteria to evaluate the clinical applicability of H‐CXI. Thus, a cohort study with registration number NCT01516944 was conducted to explore the clinical applicability of H‐CIX as a predictor of cancer cachexia and prognosis among patients with locally advanced gastric cancer (LAGC). The study prospectively enrolled 290 LAGC patients who underwent direct radical surgical resection, of whom 111 (38.28%) had complete serologic and anthropometric data. The cohort primarily composed of males (62.16%) with a mean age of 58 years. Based on the optimal cut‐off value for H‐CXI from a previous study, 61 (54.95%) patients were categorized into the high H‐CXI group and the remaining 50 (45.05%) into the low H‐CXI group. During the follow‐up period with a median duration of 64.9 months, 21 (18.92%) patients developed cancer cachexia, and patients in the high H‐CXI group had a significantly lower risk of developing cancer cachexia than those in the low H‐CXI group (11.48% vs. 28.00%, *P* = 0.027). Furthermore, patients in the high H‐CXI group showed significantly better overall survival (OS) and disease‐free survival (DFS) rates than those in the low H‐CXI group (OS: 59.02% vs. 36.00%, *P* = 0.0043; DFS: 54.10% vs. 26.00%, *P* = 0.0024). Moreover, subgroup analysis revealed better 5‐year OS and DFS rates for patients with high H‐CXI at all pathological stages. Thus, the study found that H‐CXI effectively predicted the prognosis and the risk of developing cancer cachexia in patients with LAGC, which was consistent with previous findings.

The occurrence of cachexia in cancer patients is prevalent and has a negative impact on their quality of life and treatment outcomes.^2^, ^3^ Hence, it is crucial to identify biomarkers for timely detection and evaluation of patients at high risk of developing cachexia. Several studies have demonstrated the potential efficacy of CXI as a biomarker to predict cancer patients' prognosis.^4^, ^5^ However, the practical application of CXI is limited by the associated cost and complexity of skeletal muscle index testing procedures. Sadly, recent research on developing CXI has been at a standstill. Fortunately, H‐CXI is a useful indicator that predicts cancer cachexia and prognosis effectively. Besides being convenient and quick, it provides an efficient complement to the stratification of TNM stage prognosis.

We extend our gratitude to Xie et al.^1^ for their valuable contribution and recognize the crucial implications of their discoveries for related domains. The H‐CXI method offers a comprehensive evaluation of the muscle, nutritional, and inflammatory status in LAGC patients suffering from cachexia. This method serves as a significant point of reference for the prognosis assessment and individualized treatment of patients. Our cohort study of LAGC patients reveals that H‐CXI has a high level of accuracy in predicting and assessing the cachexia and prognostication of LAGC patients. H‐CXI has the potential to enhance clinical evaluations and treatment strategies for LAGC patients, ultimately benefiting patient survival (Figure 1).

**Figure 1:**
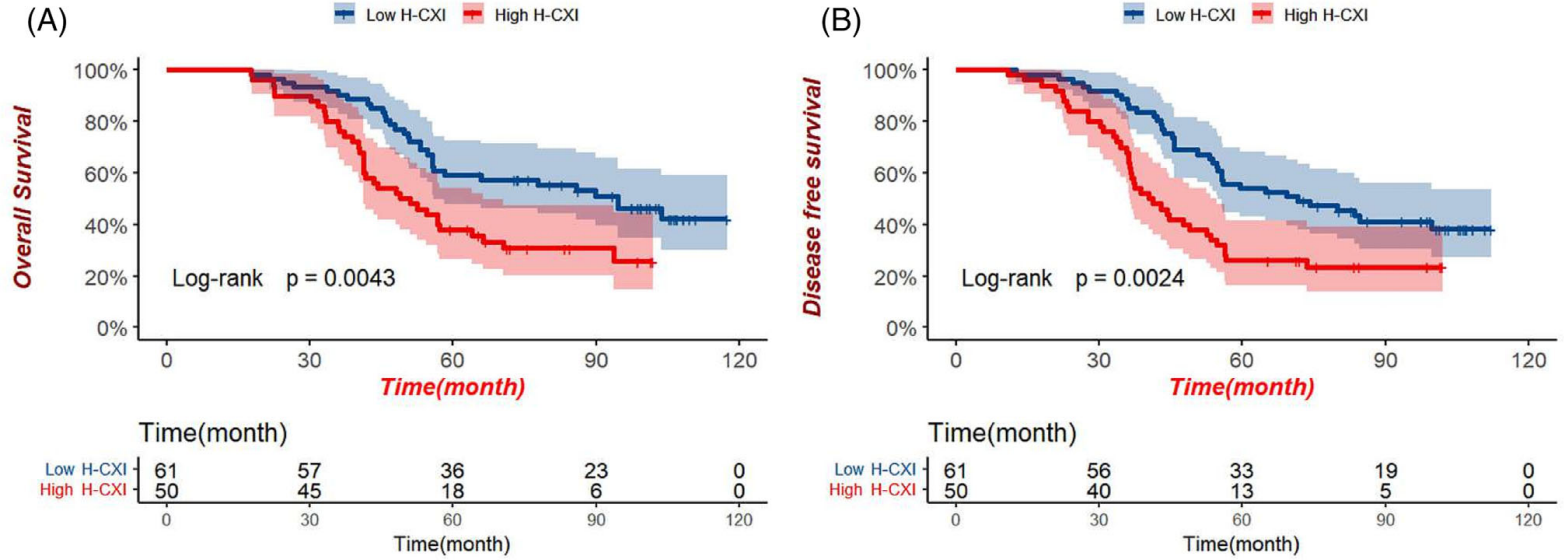
Kaplan–Meier curves of different H‐CXI levels in LAGC patients. (A) Overall survival; (B) disease free survival.

## Financial support

This work was supported by the Cultivating Outstanding Talents Project of Hebei Provincial Government Fund (No. 2019012); Hebei Public Health Committee County‐Level Public Hospitals Suitable Health Technology Promotion and Storage Project (No. 2019024); and Hebei University Science and Technology Research Project (No. ZD2019139).

## References

[1] Xie H , Ruan G , Wei L , Zhang H , Ge Y , Zhang Q , et al. Hand grip strength‐based cachexia index as a predictor of cancer cachexia and prognosis in patients with cancer. J Cachexia Sarcopenia Muscle 2023;14:382–390. 36447437 10.1002/jcsm.13139 PMC9891920

[2] da Rocha IMG , Marcadenti A , de Medeiros GOC , Bezerra RA , Rego JFM , Gonzalez MC , et al. Is cachexia associated with chemotherapy toxicities in gastrointestinal cancer patients? A prospective study. J Cachexia Sarcopenia Muscle 2019;10:445–454. 30924270 10.1002/jcsm.12391 PMC6463470

[3] Kasvis P , Vigano M , Vigano A . Health‐related quality of life across cancer cachexia stages. Ann Palliat Med 2019;8:33–42. 30525763 10.21037/apm.2018.08.04

[4] Gong C , Wan Q , Zhao R , Zuo X , Chen Y , Li T . Cachexia index as a prognostic indicator in patients with gastric cancer: a retrospective study. Cancers (Basel) 2022;14:4400. 36139560 10.3390/cancers14184400 PMC9497229

[5] Go SI , Park MJ , Park S , Kang MH , Kim HG , Kang JH , et al. Cachexia index as a potential biomarker for cancer cachexia and a prognostic indicator in diffuse large B‐cell lymphoma. J Cachexia Sarcopenia Muscle 2021;12:2211–2219. 34676685 10.1002/jcsm.12837 PMC8718032

[6] von Haehling S , Morley JE , Coats AJS , Anker SD . Ethical guidelines for publishing in the *Journal of Cachexia, Sarcopenia and Muscle*: update 2021. J Cachexia Sarcopenia Muscle 2021;12:2259–2261. 34904399 10.1002/jcsm.12899 PMC8718061

